# Transition metal anchored on red phosphorus to enable efficient photocatalytic H_2_ generation

**DOI:** 10.3389/fchem.2023.1197010

**Published:** 2023-06-14

**Authors:** Lu Lu, Mingzi Sun, Tong Wu, Qiuyang Lu, Baian Chen, Cheuk Hei Chan, Hon Ho Wong, Bolong Huang

**Affiliations:** ^1^ Department of Applied Biology and Chemical Technology, The Hong Kong Polytechnic University, Kowloon, Hong Kong SAR, China; ^2^ Research Centre for Carbon-Strategic Catalysis, The Hong Kong Polytechnic University, Kowloon, Hong Kong SAR, China

**Keywords:** red phosphorus, single-atom catalysts, transition metals, photocatalysis, H_2_ generation

## Abstract

Transition metal (TM) single atom catalysts (SACs) are of great potential for photocatalytic H_2_ production because of their abundant catalytic active sites and cost-effectiveness. As a promising support material, red phosphorus (RP) based SACs are still rarely investigated. In this work, we have carried out systematic theoretical investigations by anchoring TM atoms (Fe, Co, Ni, Cu) on RP for efficient photocatalytic H_2_ generation. Our density functional theory (DFT) calculations have revealed that 3d orbitals of TM locate close to the Fermi level to guarantee efficient electron transfer for photocatalytic performances. Compared with pristine RP, the introduction of single atom TM on the surface exhibit narrowed bandgaps, resulting in easier spatial separation for photon-generated charge carriers and an extended photocatalytic absorption window to the NIR range. Meanwhile, the H_2_O adsorptions are also highly preferred on the TM single atoms with strong electron exchange, which benefits the subsequent water-dissociation process. Due to the optimized electronic structure, the activation energy barrier of water-splitting has been remarkably reduced in RP-based SACs, revealing their promising potential for high-efficiency H_2_ production. Our comprehensive explorations and screening of novel RP-based SACs will offer a good reference for further designing novel photocatalysts for high-efficiency H_2_ generation.

## 1 Introduction

Hydrogen energy is a clean and renewable energy, attracting increasing research attention in recent years. Especially, it has a high gravimetric energy density, and the product of H_2_ combustion is only pure water, making it a promising candidate to cope with the fossil energy crisis and meet the challenge of peak carbon dioxide emission limitation. (Wang S. et al., 2019; Pan et al., 2021; Xue et al., 2022). However, the high cost of H_2_ production as well as safety issues in storage and transportation have greatly restricted its commercial applications. Without additional electric power consumption, solar energy-driven catalytic water splitting has been considered as an eco-friendly strategy for low-cost H_2_ production. Currently, photocatalytic H_2_ evolution has not been broadly adopted in large-scale industrial production since it still suffers from the low solar-to-hydrogen (STH) conversion rate, which is caused by intrinsic unfavorable thermodynamics and sluggish kinetics of solar-driven catalytic water splitting reaction. (Chen et al., 2020; Bie et al., 2022). In recent decades, to improve the catalytic activity of photocatalysts, researchers have tried a series of modulation strategies to enhance surface reactivity, reduce the activation energy barrier, and restrain the intrinsic fast recombination of photoinduced charge carriers.

Previously, phosphorus has been treated as an effective heteroatom dopant for the band structure engineering of TiO_2_-based photocatalysts. (Yu et al., 2003; Wang et al., 2012a; Hu et al., 2017; Zhu et al., 2019; Huang et al., 2022). Afterwards, it has been discovered that phosphorus itself can also act as an efficient photocatalyst for hydrogen evolution through water-splitting. (Shen et al., 2014; Hu et al., 2017; Liu et al., 2019; Chen et al., 2022). Among its three allotropes (black, P_4_-white, and red phosphorus), red phosphorus (RP) is the most available form and has excellent chemical stability at room temperature, and it has been treated as a promising semiconductor material in the construction of photocatalytic platforms. (Wang et al., 2012a; Xia et al., 2015; Hu et al., 2017; Liu et al., 2019; Chen et al., 2022; Fung et al., 2022). It has been reported that RP is a durable photocatalyst with the stable catalytic activity of H_2_ evolution for more than 90 h under visible light irradiation. (Wang et al., 2012a; Wang et al., 2012b). Though RP has good absorption of visible light at about 700 nm, the overall water-splitting efficiency of pristine RP is relatively limited due to its low conductivity, poor electron-hole separation efficiency, and sluggish charge-carrier mobility. (Zhu et al., 2019; Wang M. et al., 2020; Wu et al., 2021; Chen et al., 2022).

Since RP has good lattice compatibility, it is possible to modify the catalytic surface through the doping method or heterostructure construction. As a result, the electronic structures of RP can be well-manipulated to benefit the photocatalytic reaction. (Li et al., 2019; Zhu et al., 2020a; Huang et al., 2022; Wang et al., 2022; Jia et al., 2023). With the assistance of appropriate active co-catalyst atoms such as Pt, the overall photocatalytic H_2_ production performance of RP is greatly improved. (Wang et al., 2012a). In consideration of material cost and future industrial applications, developing non-precious metals based catalysts is of great significance for the popularization of hydrogen energy. In particular, 3d transition metals (TMs) are the most commonly studied candidates to replace noble-metal catalysts due to their high catalytic activity. By suitable tuning strategies, the novel non-noble metal catalysts can also achieve comparable or even superior catalytic activity to noble metal catalysts. Scientists have reported that both morphological and scale controls are effective for the electronic property tuning of semiconductor materials. (Zhu et al., 2020b; Wu et al., 2020; Khandelwal et al., 2022). By downsizing the material scale from the nanoscale to the cluster scale and then finally the atomic scale, an enhanced inter-atomic strain is induced, leading to the modulations in geometrical structure and electronic structures. (Liu and Corma, 2018; Khandelwal et al., 2022). Hence, developing single-atom catalysts (SACs) is a potential method for the modulation of catalytic activity on the atomic scale. The atomic dispersion of active metal atoms on the RP surface will maximumly improve the atomic utilization rate, offering maximum photocatalytic active sites to enable the light adsorption and activation of H_2_O molecules with a minimum consumption of metal atoms. (Wang B. et al., 2019; Gao et al., 2020). In previous studies, the Ni-anchored RP SACs have been proven to be very efficient photocatalysts for water splitting, with a greatly improved catalytic activity than the pristine RP. (Wang et al., 2022; Jia et al., 2023). However, the detailed photocatalytic reaction mechanisms of Ni-RP SACs and the potential of other 3d TMs SACs still lack sufficient studies.

In this work, we have conducted comprehensive calculations of the electronic structures of commonly used TMs-based SACs (TM = Fe, Co, Ni, and Cu) on RP support (denote as TMs-RP) to predict their performances of photocatalytic H_2_ generation. Based on the band structures and work functions, the thermodynamic driving force for hydrogen and oxygen evolution reaction (HER and OER) of each TMs-RP SACs is obtained and the pH tuning is also realized based on the Nernst equations. Besides, the kinetics for water adsorption and activation, the energy barrier for H adsorption, and the activation barrier of H_2_ evolution are demonstrated in detail. This work will offer an atomic scale insight into the reaction mechanisms of TMs-RP SACs based photocatalytic H_2_ evolution. Meanwhile, the detailed theoretical explorations also serve as fundamental theoretical references for the rational design of TMs-based SACs with enhanced photocatalytic performances in the future.

## 2 Calculation setup

In this work, we have conducted density functional theory (DFT) calculations within the CASTEP module. (Clark et al., 2005). The geometry optimizations and single-point energy calculations for all models have been conducted based on the GGA-PBE (Perdew et al., 1992; Perdew et al., 1996; Hasnip and Pickard, 2006) functional, ultrasoft pseudopotentials, and the BFGS algorithm. To guarantee the electronic minimization and convergence requirement, we have adopted the ensemble DFT method of Marzari et al. for the solution of the Kohn-Sham equation. (Marzari et al., 1997). The cutoff energy has been applied with the ultrafine quality, which is set as 380 eV for RP and Fe-RP, Co-RP, Ni-RP, and 440 eV for Cu-RP. The k-point set is 2 × 2×1 for all the energy minimizations. The convergence parameters are set up as follows: the SCF tolerance is 5.0 × 10^−7^ eV/atom; the Max. Hellmann-Feynman force per atom is 0.01 eV/Å; the Max. stress is 0.02 GPa; and the Max. displacement is 5.0 × 10^−4^ Å. According to previous experimental studies, the [001]-oriented Hittorf’s phosphorus has been proven to be very efficient for water-splitting. (Zhu et al., 2020a). Thus, in this work, we choose the RP (001) surface to act as the supporting host to stabilize the single-atom (SA) TMs. The RP (001) facet is built from relaxed bulk Hittorf’s phosphorus, (Thurn and Krebs, 1966), with a thickness of two layers including 84 atoms (Supplementary Figure S1A). The top view of pristine RP (001) and TMs-RP (Fe, Co, Ni, and Cu) catalysts are demonstrated in Supplementary Figures S1B–F. The lattice parameter is 9.27 Å and 9.21 Å for length A and B-orientations, respectively. The vacuum thickness is set to be 20 Å, resulting in a length of 40.95 Å in C-orientation to ensure sufficient space for geometry optimizations. During the adsorption of H_2_O and H on different TMs-RP in this work, the SAC surfaces have been constrained in order to highlight the behaviors of key adsorbates. The H-adsorption free energy (ΔG_H*_) serves as a key descriptor for the prediction of HER activity, which can be calculated based on the following equation (Nørskov et al., 2005; Kerketta et al., 2022):

$$\Delta E_{H}=E_{H^{*}}-E^{*}-1/2½E\left(H_{2}\right)$$ (1)

In this equation, * indicates the un-adsorbed pure surface, and H* stands for the surface adsorbed with H.

## 3 Results and discussions

### 3.1 Electronic structures of TMs-RP

For photocatalytic reactions, the excitation energy required for electron-hole separation is largely decided by the bandgap value of the semiconductor photocatalyst. To make the most use of solar energy, the bandgap of the photocatalysts is expected to be located within the spectrum of sunlight, namely, from the UV to NIR range. In fact, most of the solar energy (nearly 95%) is constituted by visible light (43%) and NIR (52%). To match the Vis-NIR excitation window of sunlight, the bandgap of the candidate photocatalyst is expected to be less than 3.10 eV. And based on the redox potential of water, to drive the water splitting reaction, the bandgap value is projected to be wider than 1.23 eV with the location of VBM lower than the oxidation level of O_2_/H_2_O and CBM higher than the reduction level of H_2_/H^+^. Thus, the bandgap values and band positions of photocatalysts can largely reflect their absorption window toward sunlight, and the relative positions of VBM and CBM with reference to standard hydrogen electrode potential can be used to predict the redox tendency for water splitting.

**FIGURE 1:**
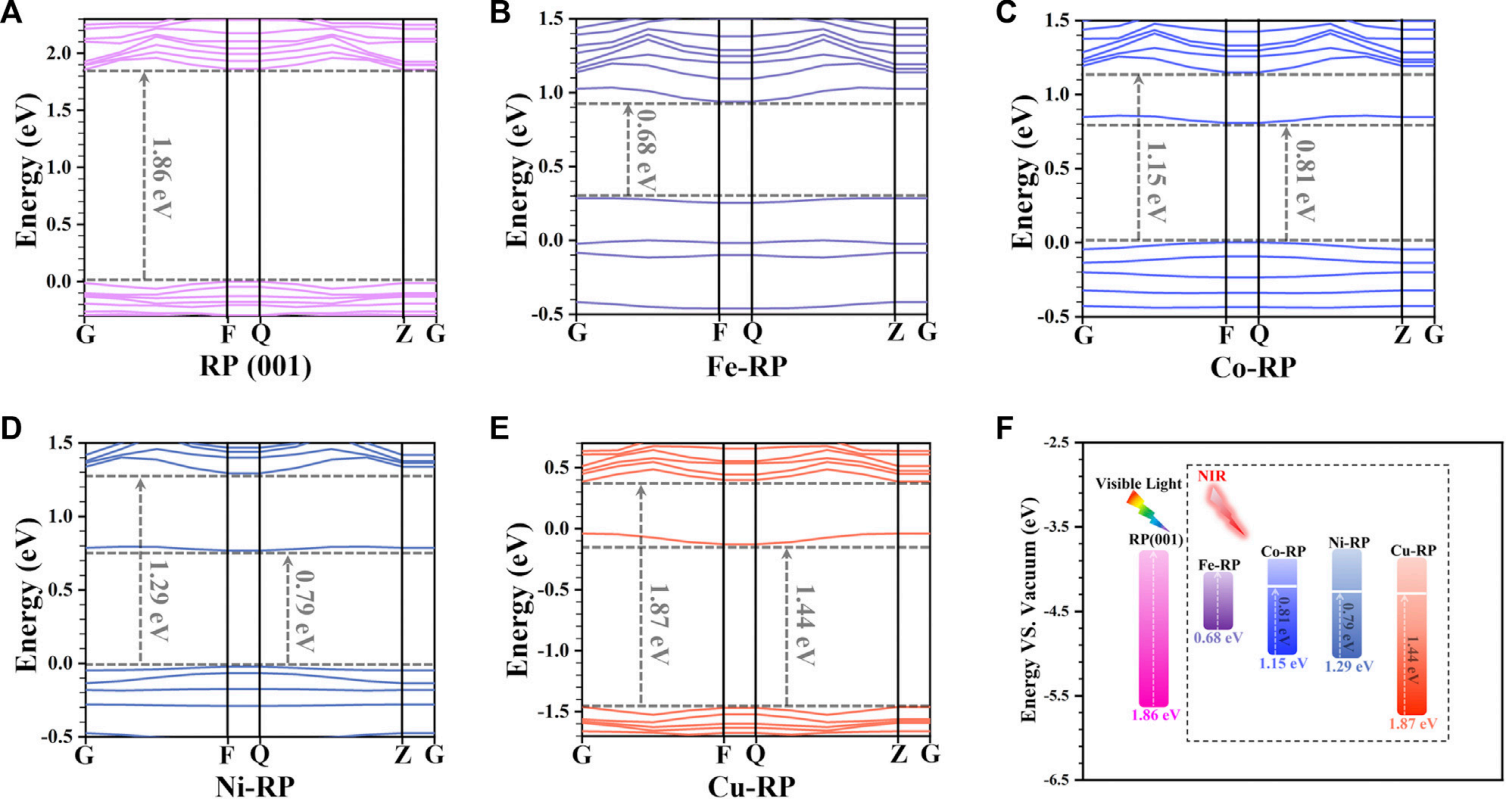
Band structures of **(A)** RP (001), **(B)** Fe-RP, **(C)** Co-RP, **(D)** Ni-RP, and **(E)** Cu-RP, respectively. **(F)** Summarized bandgap positions and values for pristine-RP (001) as well as Fe-, Co-, Ni-, and Cu-anchored RP.

To evaluate the bandgap matching degree of the TMs-RP, we have first compared the band structure of pristine RP (Figure 1A) with Fe-, Co-, Ni-, Cu-anchored RP, respectively (Figures 1B–E) to reveal the anchoring effect from different TM atoms. For the pristine RP (001) surface, the bandgap is calculated to be 1.86 eV, corresponding to an excitation energy of 667 nm in the red-light range. For Fe-, Co-, and Ni-anchored RP, we find additional TM states generated upper to the valence band maximum (VBM) and below the conduction band minimum (CBM), resulting in a narrowed bandgap of 0.68 eV, 1.15 eV, and 1.29 eV, respectively. Notably, the bandgap of Cu-RP is not affected by Cu single atoms, which still remains 1.87 eV. In particular, owing to the states appeared below CBM in Co-RP (0.81 eV) and Ni-RP (0.79 eV), the excitation energy required for the generation of photon-induced charge carriers has largely shifted to the NIR range (1531 nm–1824 nm), as indicated in Figure 1F. For Cu-RP, there is an obvious downshifting of both VB and CB, which shows only one mid-gap state with a distance of 1.44 eV from the VBM, which reduces the absorption energy to a lower NIR window (861 nm).

**FIGURE 2:**
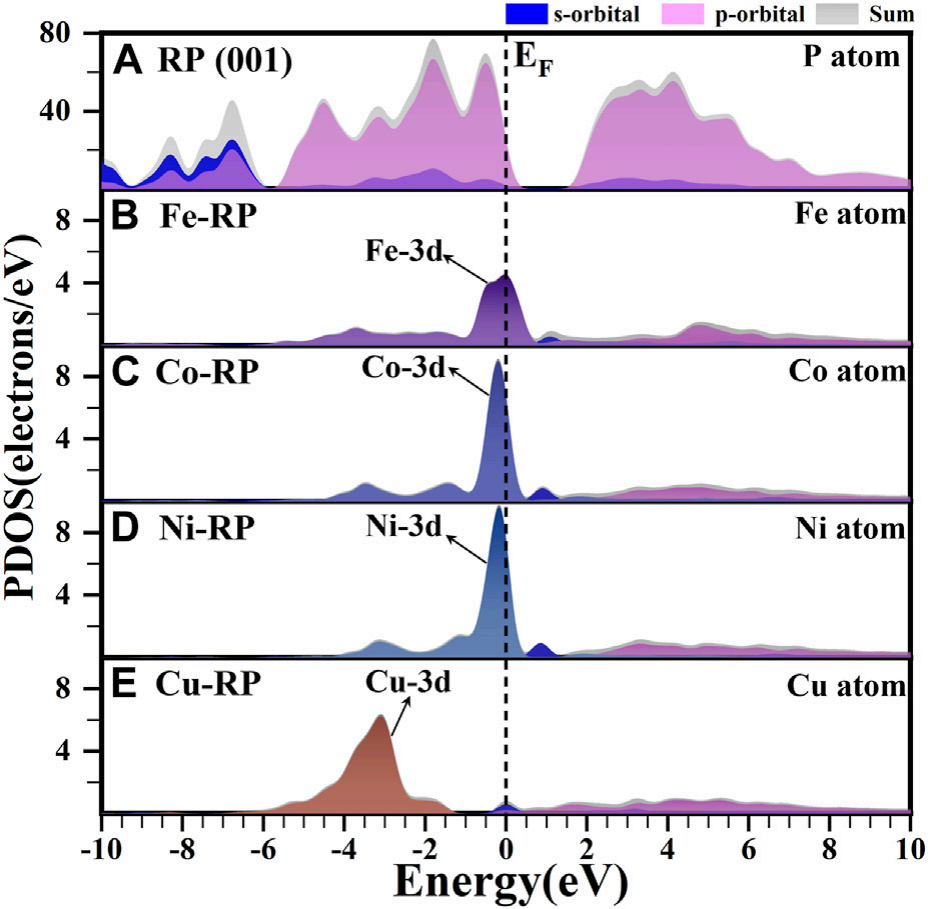
The calculated PDOS results of **(A)** P atom in RP (001), **(B)** Fe atom in Fe-RP **(C)** Co atom in Co-RP, **(D)** Ni atom in Ni-RP, and **(E)** Cu atom in Cu-RP respectively. The dashed line means the Fermi level.

Besides band structures, the calculated partial density of states (PDOS) is another important indicator of electronic structures. In Figure 2, we focus on the analysis of the d-orbital property of each TM since the d-band center is also strongly related to the photocatalytic activity and the electron transfer efficiency. (Zhou et al., 2022). The pristine RP shows an evident bandgap between CBM and VBM, which are both dominated by the P-3p orbitals (Figure 2A). For Fe-, Co-, and Ni-anchored RP, the localized d orbitals locate very close to the Fermi level (E_F_) and VBM (Figures 2B–D). Especially, Co-RP and Ni-RP both display very sharp 3d orbitals with higher electron density near E_F_, exhibiting better performance for electron transmission. In contrast, the 3d orbitals of Cu-RP have significantly moved to a lower position of VB, which is far from the E_F_ (Figure 2E). This results in a wider gap for the transition of photo-induced charge carriers during photocatalysis. The band edge is mainly contributed by the p-orbitals of P with overlapping of 3d orbitals of TMs, which induces the p-d coupling effect to further facilitate charge-carrier transfer. For all four TMs-RP catalysts, we notice the contribution of s-orbital to the CBM, which serve as a ladder to lower the excitation energy for electron excitation from VB to CB. For Cu-RP, the s-orbital dominates the E_F_, which only limitedly compensates for low electron transfer density near the VBM. Overall, the PDOS results have revealed that the single-atom TM anchored in RP, especially Co- and Ni-, brings great improvements in both separations and transportations of photo-generated charge carriers during photocatalysis.

### 3.2 Band structure alignment analysis

**FIGURE 3:**
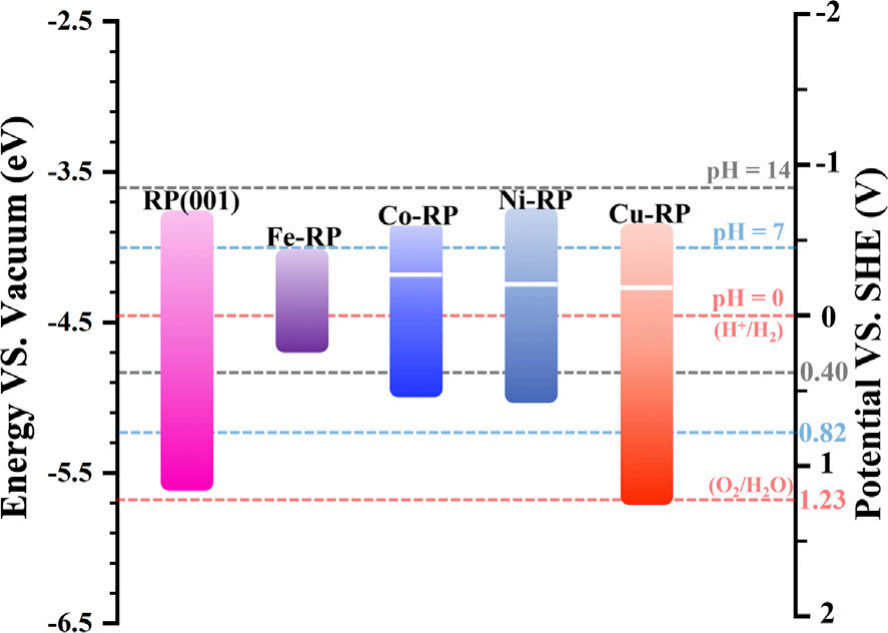
Band structure alignments in the scale of Vacuum (left) and SHE (right) for pristine-RP (001), Fe-RP, Co-RP, Ni-RP, and Cu-RP, with reference to the redox pairs of H^+^/H_2_ and O_2_/H_2_O. Red dashed lines indicate pH = 0, blue dashed lines represent pH = 7, and black dashed lines stand for pH = 14.

The electron-transition capacity of valence electrons is decided by the energy gap between E_F_ and vacuum level. (Zhou et al., 2022). Thus, the work function has been treated as a key descriptor for electron transition and charge flow in heterojunction structure, and it can be obtained from the calculated electrostatic potential. (Qin et al., 2020; Ruan et al., 2022). As discussed above, besides the bandgap values, the relative position of the bandgap with a reference to standard hydrogen electrode potential is also critical to predict photocatalytic activity. For the construction of the band alignment diagram (Figure 3), the electrostatic potential for each TM-RP type studied in this work has been calculated to indicate the distance between the Fermi level and vacuum level (Supplementary Figures 2A–E). The thermodynamic driving force for photocatalysis is mainly affected by the relative potential position of CBM and VBM of the semiconductor as well as the redox potential of the corresponding reversible reaction. (Li et al., 2016). More negative CBM means stronger reduction reaction tendency, while more positive VBM potential signifies stronger oxidation reaction driving force. Therefore, besides a bandgap value of more than 1.23 eV, the band edges (both VBM and CBM) of the photocatalyst candidates need to cover both the standard potentials of OER and HER to guarantee the high performances of water-splitting. (Mortazavi et al., 2021).

In the acidic condition (pH = 0 in aqueous solution), the CBM of pristine RP and TM-anchored RP are all located above the reduction level of H_2_/H^+^, exhibiting a high hydrogen evolution tendency (Supplementary Figure S3). However, based on the energy gap between CBM and H_2_/H^+^ level, the TMs-RP all exhibit a relatively weaker hydrogen evolution tendency than the pristine RP surface except Ni-RP. According to the large gap value from Ni-RP CBM to the H_2_/H^+^ level, we propose that the anchoring Ni atom offers a stronger driving force for the hydrogen evolution reaction. Meanwhile, the VBM positions of pristine RP and TM-RP (except Cu-RP) are located above O_2_/H_2_O level, theoretically indicating the weak capability for oxygen evolution. Previous works have revealed the slight mismatching of bandgaps and work functions between theoretical calculations and experimental data. (Wang et al., 2009; Wang et al., 2012a). By considering the calculated SHE as a reference, the pristine RP and Cu-RP are also able to achieve oxygen evolution in the acidic condition.

Since standard redox potentials of H_2_O are sensitive to pH variation, the photocatalytic driving force for HER and OER can be well tuned by the pH manipulation based on the following Nernst equations: (Shojaei et al., 2020; Mortazavi et al., 2021):

$$E^{HER}\left(H^{+}/H_{2}\right)=-4.44\;eV+pH\times 0.059\;eV$$ (2)

$$E^{OER}\left(O_{2}/H_{2}O\right)=-5.67\;eV+pH\times 0.059\;eV$$ (3)

The pH variation also impacts the band positions, which has been verified in experiments that the slope of E_CB_ (−0.033 eV/pH) does not follow the Nernstian dependence on pH (−0.059 eV/pH). (Simon et al., 2014). The slope of Nernstian dependence on pH for H_2_O redox potentials is steeper. However, in this work, the pH value influences on the corresponding band positions are not included. In our work, the model is built in a vacuum environment with the mainly related elements (H_2_O, H, or OH) adsorbed on the catalytic surface in HER. The corresponding band positions are stationary with redox potentials of H_2_O in different pH values to reveal the reaction tendency for HER and OER. (Zhang et al., 2021; Yang et al., 2018; Makaremi et al., 2018; da Silva et al., 2019; Shahid et al., 2020). In the neutral condition (pH = 7), the H_2_/H^+^ level and O_2_/H_2_O level move up to a more negative position (with reference to SHE) at −0.41 eV and 0.82 eV, respectively (Supplementary Figure S4). Accordingly, for the pristine RP and TMs-RP photocatalysts, the driving force for HER declines while the tendency for OER increases. RP and Cu-RP still cover both potential levels of HER and OER, indicating their photocatalytic activity towards the full water splitting under neutral conditions. In addition, in the highly alkaline environment (pH = 14), pristine RP and TMs-RP nearly lose their HER capability in terms of thermodynamics (Supplementary Figure S5). Thus, we can predict that in the design of photocatalytic heterojunction, Ni-RP is a possible candidate to serve as an efficient HER photocatalyst in wide pH environments.

### 3.3 Water adsorption comparison

Besides thermodynamic driving force, many other factors such as microstructures at the micro or nanoscale, adsorption energy, surface/interface morphological properties, and coupling effects with cocatalyst components also have significant influence on photocatalytic performance. (Li et al., 2015). For a better explanation of the reaction mechanism and the prediction of the catalytic activity of a potential photocatalyst, the adsorption of reactants and the reaction energy should also be included. The complex charge-carrier dynamics as well as surface-reaction interactions should be fully investigated since they can largely affect the apparent quantum efficiency in multi-stage heterogeneous photocatalytic reactions. (Li et al., 2016).

The H_2_O adsorption energy has a great influence on interfacial charge transfer, and decides the aggregation extent of H_2_O molecules near the catalytic active sites, which further triggers the concentration effect and results in more H* generated from water splitting. (Wang F. et al., 2020). The adsorption strength of H_2_O molecules is greatly affected by the structural stability of single atoms anchored catalytic surface. In particular, the interactions between the anchored SA-TMs and supporting P atoms have been investigated regarding the iso-surface of charge density difference (Supplementary Figure S6) and the Mulliken charge (Supplementary Table S1). The RP stabilized SA-atoms have an obvious electron exchange with coordinated P atoms. Notably, we discover that the Fe atoms (0.27 e) have a stronger interaction with surrounding P atoms than those of Co-RP (0.24 e) and Ni-RP (0.18 e) counterparts. The stable anchoring site on the RP surface of SA-Cu is different from the other three TMs-RP, which is not in the center of the hexatomic ring formed by P atoms. SA-Cu shows a relatively larger average net charge loss of 0.30 e. The electron flow between the stabilized SA-TM atoms and supporting atoms indicates the modification of electronic structures on the RP surface, which verifies the feasibility of doping TMs for photocatalytic activity modulation.

**FIGURE 4:**
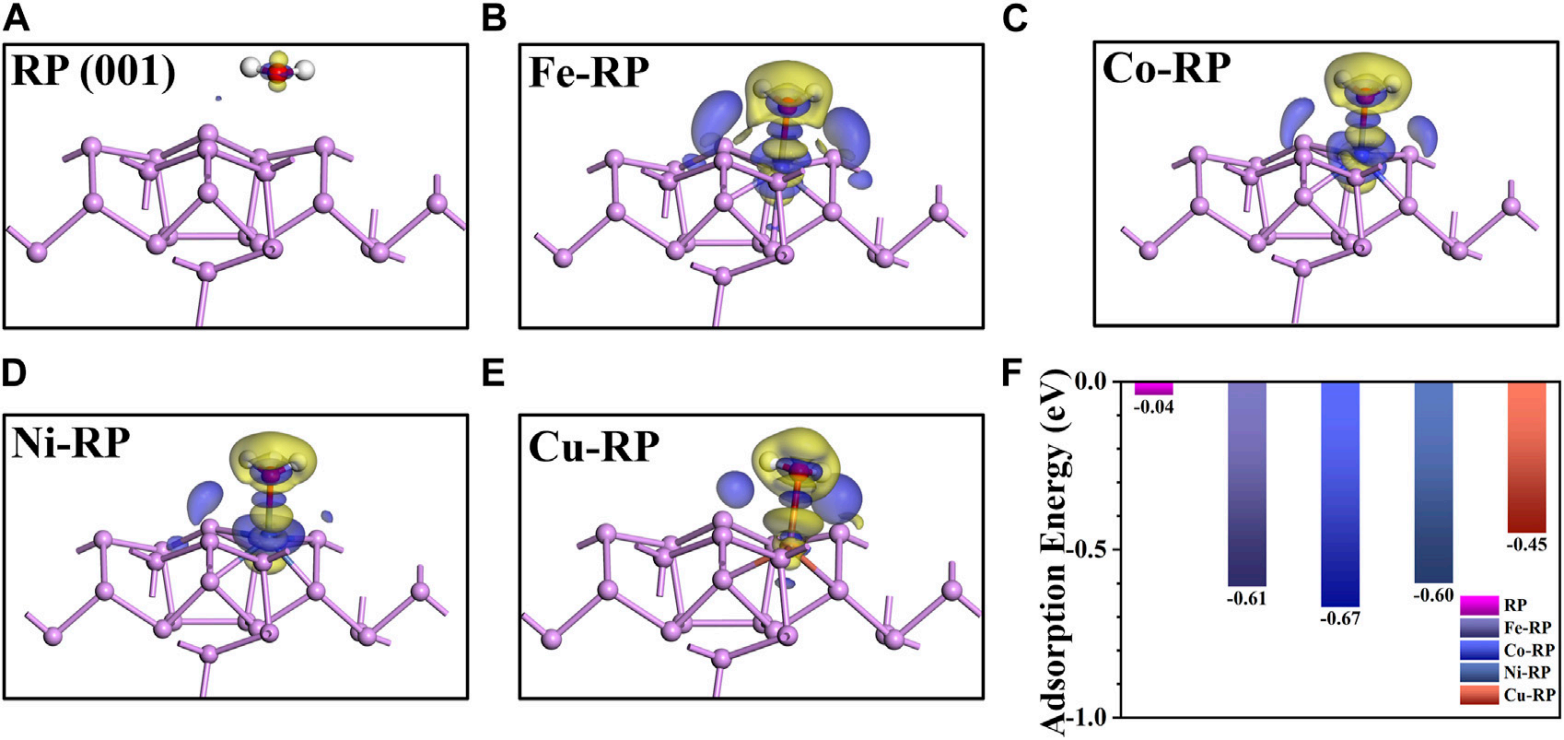
The charge density difference for the net flow of charge between active P/TM atom and O atom in H_2_O for **(A)** RP (001), **(B)** Fe-RP, **(C)** Co-RP, **(D)** Ni-RP and **(E)** Cu-RP. The blue color and red color mean the gain and loss of electrons, respectively. **(F)** The comparison of water adsorption energy.

From the charge density difference diagram in Figures 4A–E **,** H_2_O molecules have a much stronger interaction with TMs-RP when compared to pristine RP surfaces. We can see the electron loss in active metals SA sites and adsorbed O atoms (from adsorbed H_2_O). Then, the electrons aggregate between TM and H_2_O with the formation of an obvious adsorption bond. According to the calculated H_2_O adsorption energy values in Figure 4F, H_2_O adsorption processes in pristine-RP and TMs-RP are all exothermic, indicating spontaneous adsorption trends. By the loading of TMs SA atoms on the RP surface, the water adsorption capacity has been improved remarkably. Among the four TM-based SACs, Fe-RP (−0.61 eV), Co-RP (−0.67 eV), and Ni-RP (−0.60 eV) exhibit better H_2_O affinity than Cu-RP (−0.45 eV). If the H_2_O adsorption is too strong, the over-adsorption induces poison effects on the catalytic surface. (Sravan Kumar et al., 2020). Overall, the adsorption energies of Fe-, Co-, Ni-, and Cu- RP are not too negative, indicating less possibility for catalyst poisoning effect during the photocatalysis.

### 3.4 Reaction energy change of H_2_ generation

**FIGURE 5:**
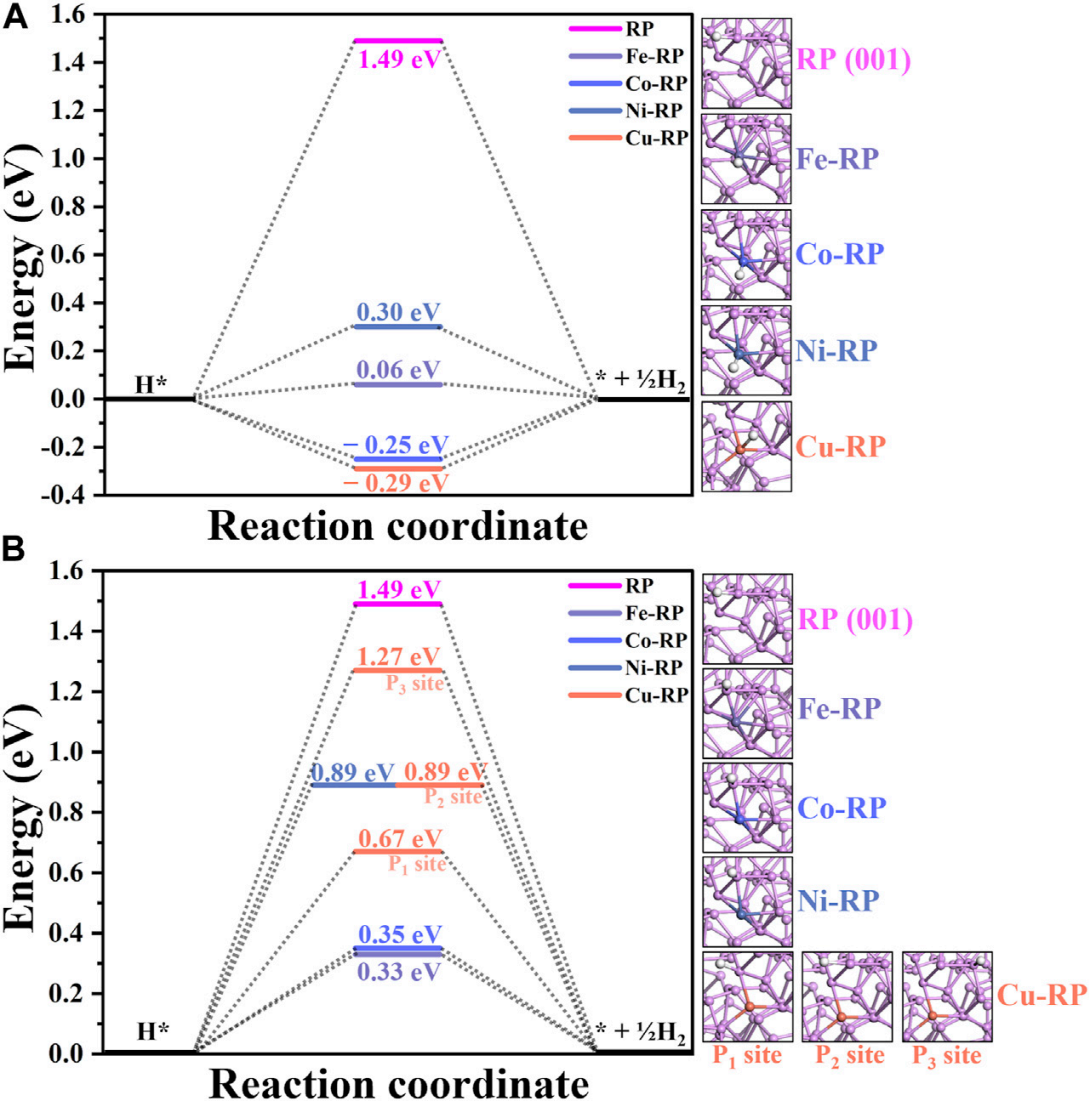
The activation barrier for H adsorption on RP (001) and single atom TM anchored RP surface: **(A)** H adsorbed on transition metal atoms. **(B)** H adsorbed on coordinated P atoms.

Besides the water adsorption comparisons, H adsorption is another determinant factor in HER, where both too negative and too positive values are not beneficial for the HER. Based on the adsorption sites, the calculations for H-adsorption reaction energy change are classified into two different types (Figure 5). One considers that H atoms are adsorbed on the SA-metal atoms (Figure 5A), and the other one demonstrates the adsorption of H atoms on the coordinated P atoms to the SA-metal atom (Figure 5B). To guarantee satisfactory computational accuracy, consistent calculation parameters have been set for all the models. For the situations that H atoms adsorbed on SA-metal atoms (H-TMs), the 4 TM SA (Fe-, Co-, Ni-, and Cu-) anchored RP catalysts all have much smaller energy barriers (0.06 eV, −0.25 eV, 0.30 eV, and −0.29 eV, respectively) than that of pristine RP (1.49 eV). As for H adsorbed on coordinated P atoms (H-P-TMs), it can be discovered that the energy barriers of H-P-TMs are slightly larger than those in H-TMs, 0.33 eV (Fe-RP), 0.35 eV (Co-RP), 0.89 eV (Ni-RP), 0.67 eV (Cu-RP, P_1_ site), 0.89 eV (Cu-RP, P_2_ site), and 1.27 eV (Cu-RP, P_3_ site) respectively, but their calculated Δ*E*_H_ values are still much less than that of pristine-RP. To sum up, under an acidic environment, the H* is mainly from H^+^ in the solution, where the adsorption energy of H* on the catalyst surface is the main influence factor for HER. Based on the calculated Δ*E*_H_, in both situations, H adsorbed on TM atoms (Fe-RP < Co-RP < Cu-RP < Ni-RP < RP) or on coordinated P atoms (Fe-RP < Co-RP < H-P_1_-Cu < Ni-RP = H-P_2_-Cu < H-P_3_-Cu < RP), SA TMs-RP have much lower energy barriers than pristine-RP for HER. It is worth noting that in the acidic condition, Fe-RP exhibits the lowest energy HER barrier in both H-adsorption sites discussed above.

**FIGURE 6:**
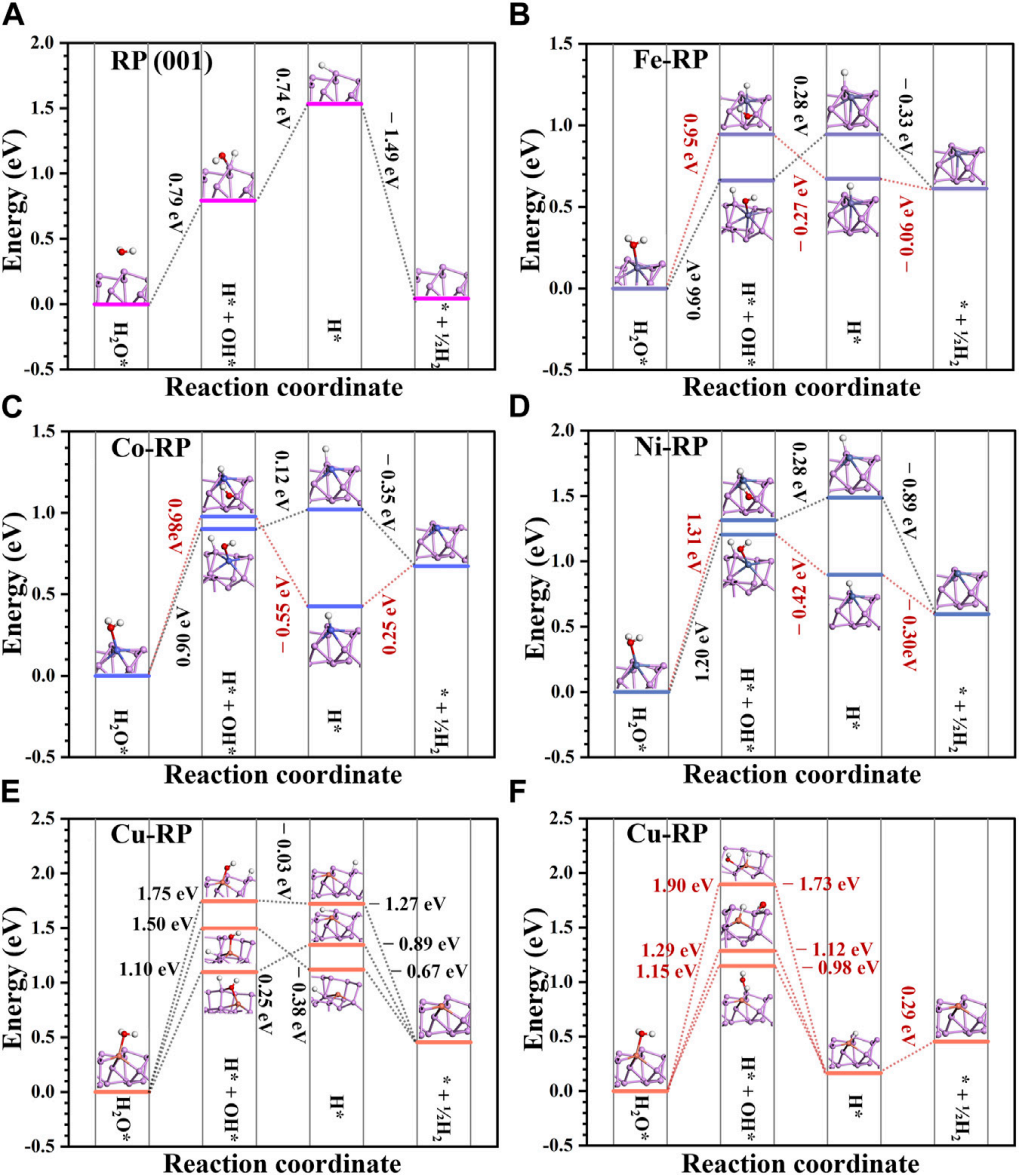
The reaction energy change of hydrogen generation on **(A)** RP (001), **(B)** Fe-RP, **(C)** Co-RP, **(D)** Ni-RP, and **(E–F)** Cu-RP surface. The black dashed lines labeled reaction paths indicate the OH adsorbed on TM atoms and H adsorbed on neighboring P atoms while the red dashed lines labeled paths demonstrate OH adsorbed on coordinated P atoms and H adsorbed on TM atoms during the water dissociation process.

For the photocatalytic mechanism in neutral and alkaline environments, H* is mainly from water dissociation. The energy barrier for the dissociation of H_2_O molecules and the desorption of OH^−^ are important influence factors in reaction energy changes. As shown in Figures 6A–F, the energy change for the whole reaction towards H_2_ generation is compared. For all the catalysts, the water dissociation step requires the largest energy costs as the rate-determining step. We have considered two different situations for water dissociation to compare the reaction energy barriers. For the adsorption of OH and H on TM atoms and neighboring P atoms, respectively, the Co-RP and Ni-RP show an energy barrier of 0.90 eV and 1.20 eV, respectively. Different from other TMs-RP types, the anchored single-atom Cu is not symmetrically located in the hexatomic ring of supporting P atoms, which leads to three possible P sites for water dissociations with a water dissociation energy of 1.50 eV, 1.10 eV, and 1.75 eV, respectively (Figure 6E). For water dissociation, Co-RP, Ni-RP, and Cu-RP exhibit higher energy barriers than that of pristine RP (0.79 eV). Notably, Fe-RP shows the smallest energy barrier (0.66 eV) during water dissociation process among all the candidate catalysts. After H_2_O dissociation, if the OH is adsorbed on coordinated P atoms and H is adsorbed on TM atoms, the activation energy barriers needed are relatively higher than the former path discussed above, indicating a lower HER efficiency. While the following desorption of OH^−^ becomes much easier on the TMs-RP surface, which requires much lower energy barriers (<0.28 eV) or even becomes spontaneous. In comparison, the pristine RP exhibits a much higher energy cost for the OH^−^ desorption, which leads to the poisoning effect of active sites and lowers the overall photocatalysis performances. Moreover, only Cu-RP and Co-RP (H adsorbed on Co atoms) show endothermic trends for the direct H_2_ formation step while other TMs-RP all show the exothermic trend.

For TMs-RP, the energy barriers for OH* desorption are relatively small. In neutral and alkaline environments, since the H* is mainly from the dissociation products (H* and OH*) of adsorbed H_2_O* molecules, the water adsorption energy and dissociation energy are the two main influencing factors for HER. The water adsorption energy follows the order of Co-RP < Fe-RP < Ni-RP < Cu-RP < RP, while the water dissociation energy, which is the largest energy barrier, follows the order of Fe-RP < RP < Co-RP < Cu-RP < Ni-RP. Less H_2_O molecules tend to be gathered around the active catalytic sites on pristine RP surface because its adsorption energy is only −0.04 eV. Thus, although pristine-RP has a relatively small energy barrier for water dissociation, its HER activity will be limited due to the inadequate gathering of reactant H_2_O molecules on the catalytic surface. The adsorption energies of water molecules on Fe-RP, Co-RP, and Ni-RP are much lower than that of pristine-RP and Cu-RP, leading to more efficient accumulation of H_2_O molecules on these three catalyst surfaces, which will benefit the following water dissociation process. Further taking the order of water dissociation energy into consideration, the activation barriers of Ni-RP and Cu-RP are relatively larger. Overall, in neutral and alkaline conditions, the Fe-RP and Co-RP can potentially offer superior HER performance.

Combining the band alignment and reaction energy, we have proposed photocatalyst candidates for different environments. For the acidic solution, Fe-RP, Co-RP, and Cu-RP are the most promising candidates. For the neutral environments, Fe-RP delivers better performances than other TMs-RP, indicating novel selections to achieve even superior performances than the reported Ni-RP catalysts. (Wang et al., 2022; Jia et al., 2023). In a weak alkaline environment, only Ni-RP is possible to satisfy the requirement of band alignments. As we further consider the electronic structure analysis, the much larger bandgap of RP and Cu-RP significantly lowers the charge separation and transfer process after light excitation, which largely affects their photocatalysis performances. Therefore, we propose that Fe-RP and Co-RP are promising candidates for photocatalysis of H_2_ generation.

## 4 Conclusion

In this work, we have conducted comprehensive investigations on photocatalytic activity regarding the electronic properties, adsorption properties, and reaction energy change for four kinds of SACs by anchoring TM (Fe, Co, Ni, and Cu) single atoms on RP. With the introduction of the single-atom TM anchored on the surface, the sunlight absorption window is extended from red light to a higher NIR region, which potentially improves the utilization rate of solar energy. The electronic structures indicate that Co-RP and Ni-RP show highly catalytic active 3d orbitals to benefit the electron transfer during photocatalysis. Considering the band alignments of photocatalysts, Ni-RP indicates its superiority in supporting HER capability over a wide pH range. Under the acidic environment, Cu-RP, Fe-RP, and Co-RP demonstrate the most preferred proton binding to promote H_2_ generation. For the neutral and alkaline solution, all TMs-RP catalysts exhibit much stronger H_2_O adsorption than pristine RP, which promotes the following dissociation to supply sufficient proton. In particular, Fe-RP and Co-RP has shown the lowest energy barriers for H_2_ generation. Overall, we notice that there is no TMs-RP that has superiority in all pH environments, where different TMs have their specific merits. Based on the comprehensive investigations of different parameters, we think that the TMs-RP possesses great potential and flexibility to achieve efficient H_2_ generation in different environments. This work has supplied important theoretical references and opened great opportunities for experimental researchers to further optimize the photocatalytic activity in advanced and novel SACs.

## Data Availability

The original contributions presented in the study are included in the article/Supplementary Material, further inquiries can be directed to the corresponding author.
