# Supplementary material for: Transition metal anchored on red phosphorus to enable efficient photocatalytic H2 generation
Source: Front Chem. 2023 Jun 14;11:1197010. doi: 10.3389/fchem.2023.1197010 (PMC10305857; doi:10.3389/fchem.2023.1197010)
Supplement: Supplementary file 1 [file DataSheet1.docx]

Supplementary Material

Transition Metal Anchored on Red Phosphorus to Enable Efficient Photocatalytic H_2_ Generation

Lu Lu ^1^, Mingzi Sun^1^, Tong Wu^1^, Qiuyang Lu^1^, Baian Chen^1^, Cheuk Hei Chan^1^, Hon Ho Wong ^1^ and Bolong Huang ^1,2^*

*** Correspondence:** Bolong Huang*: [bhuang@polyu.edu.hk](mailto:bhuang@polyu.edu.hk)

# Supplementary Figures

**
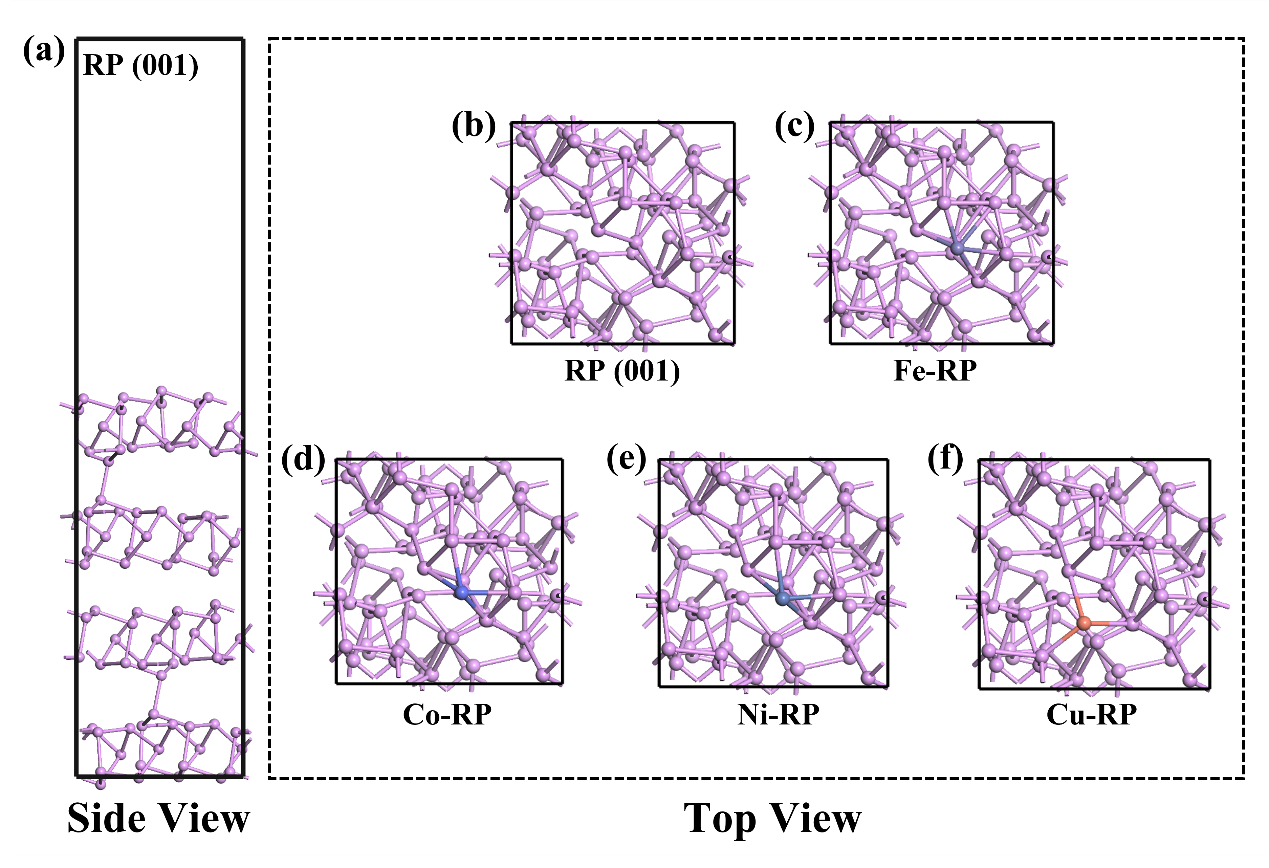
**

**Supplementary Figure 1.** (a) The side view of RP (001). The top view of (b) RP (001), (c) Fe-RP, (d) Co-RP, (e) Ni-RP, and (f) Cu-RP respectively. The magenta balls indicate P atoms, the violet sphere is the Fe atom, the blue sphere is the Co atom, the grey sphere is the Ni atom, and the orange sphere is the Cu atom.


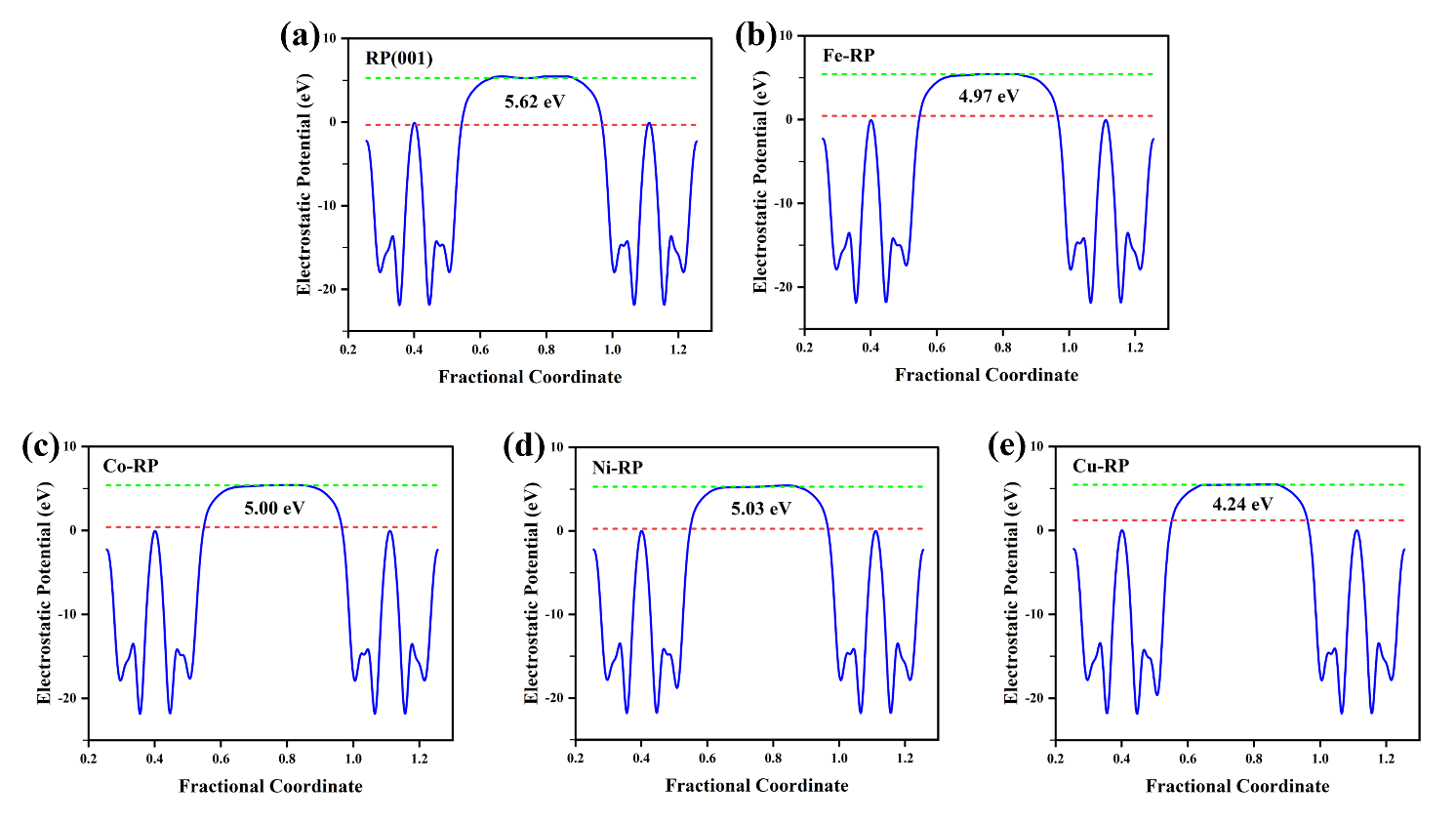


**Supplementary Figure 2.** The electrostatic potential of (a) RP (001), (b) Fe-RP, (c) Co-RP, (d)Ni-RP, and (e) Cu-RP respectively; the green dashed line represents the vacuum level, and the red dashed line represents the Fermi level.


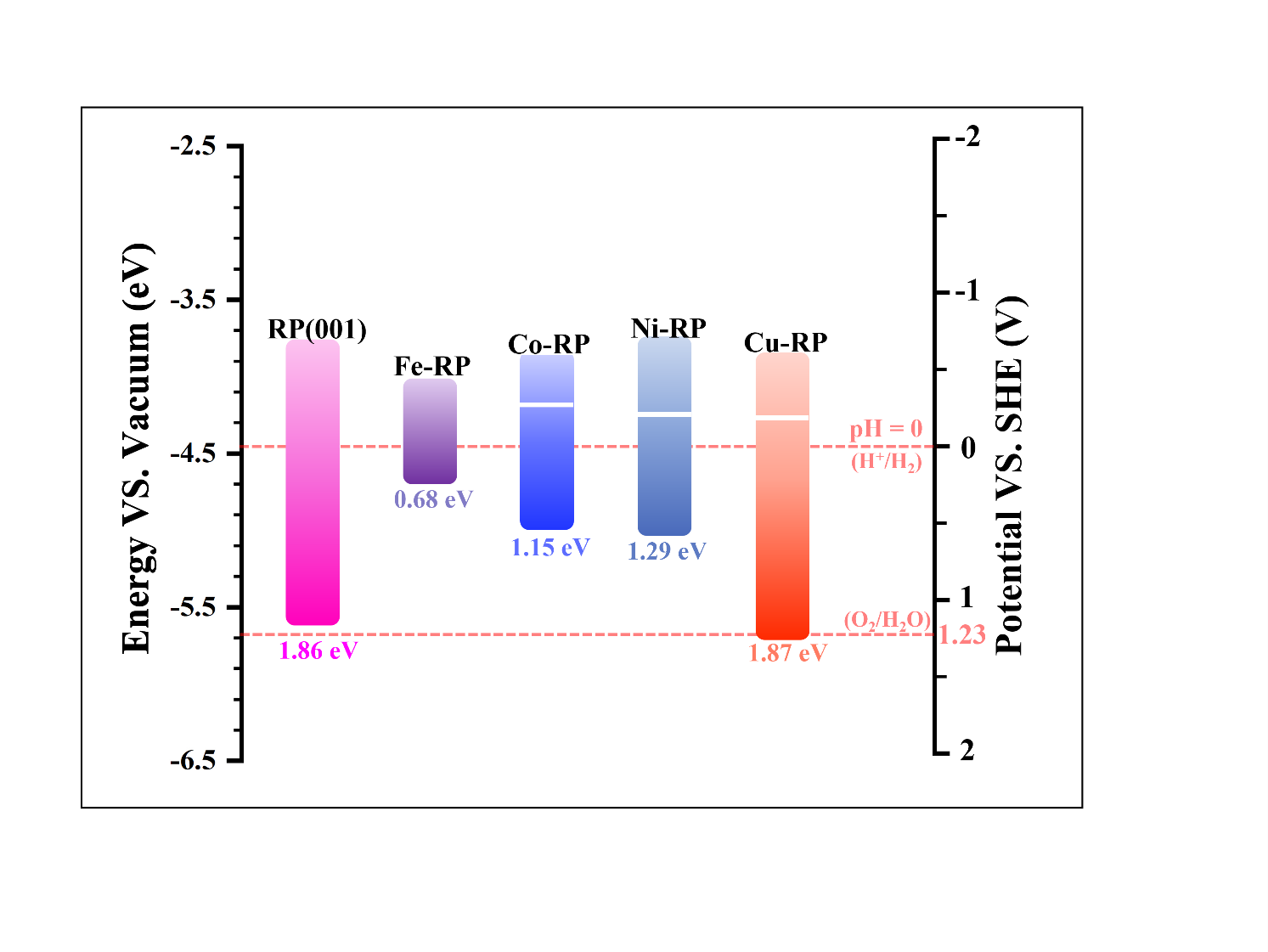


**Supplementary Figure 3.** Band structure alignments in the scale of Vacuum (left) and SHE (right) for pristine-RP (001), Fe-RP, Co-RP, Ni-RP, and Cu-RP under pH=0, with reference to the redox pairs of H^+^/H_2_ and O_2_/H_2_O.

**HER Candidates: RP, Fe-RP, Co-RP, Ni-RP, and Cu-RP**


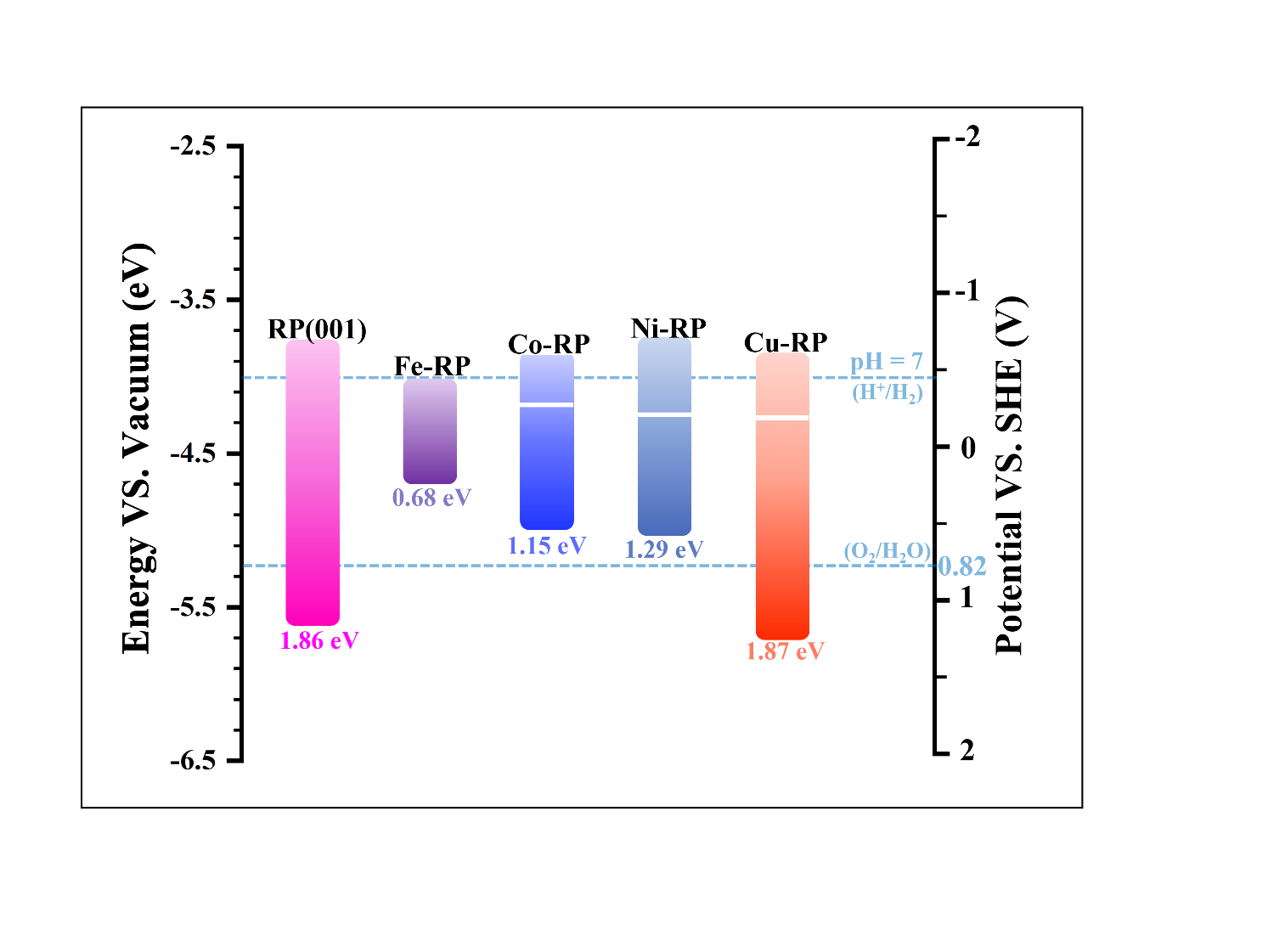


**Supplementary Figure 4.** Band structure alignments in the scale of Vacuum (left) and SHE (right) for pristine-RP (001), Fe-RP, Co-RP, Ni-RP, and Cu-RP under pH=7, with reference to the redox pairs of H^+^/H_2_ and O_2_/H_2_O.

**HER Candidates:** RP and Ni-RP still have good HER tendency. The reaction tendency of Co-RP and Cu-RP have reduced a lot, and the HER potential of Fe-RP becomes very weak.


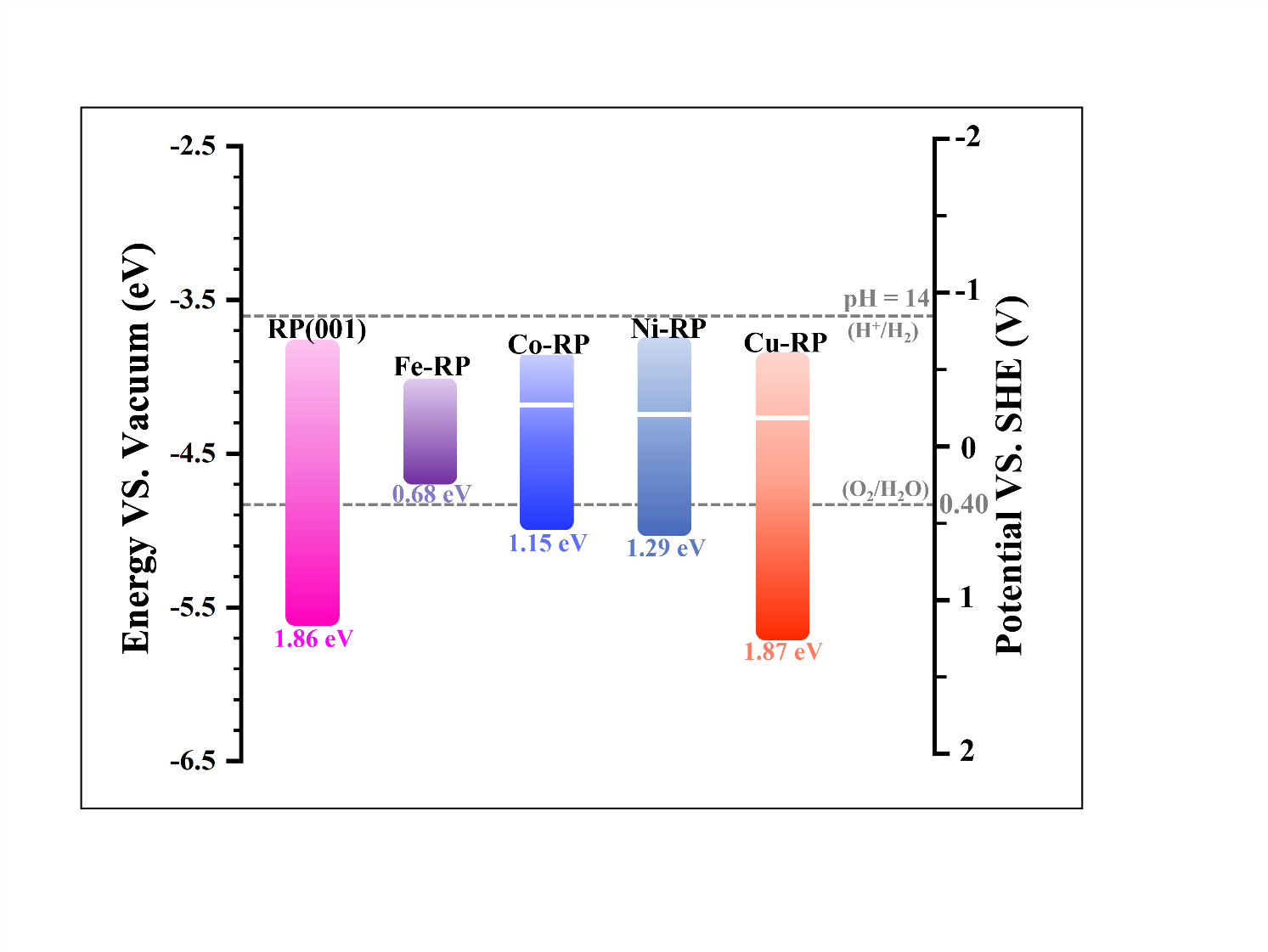


**Supplementary Figure 5.** Band structure alignments in the scale of Vacuum (left) and SHE (right) for pristine-RP (001), Fe-RP, Co-RP, Ni-RP, and Cu-RP under pH=14, with reference to the redox pairs of H^+^/H_2_ and O_2_/H_2_O.

**HER Candidates:** Pristine RP and TMs-RP lose their HER activity.

**
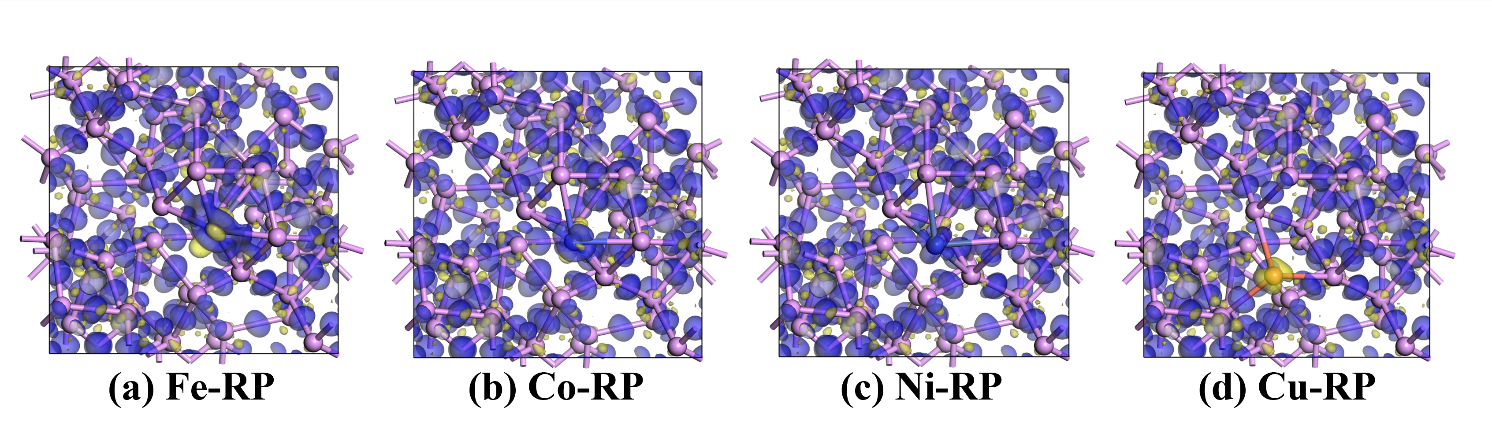
**

**Supplementary Figure 6.** The iso-surface of charge density difference of (a) Fe-RP, (b) Co-RP, (c) Ni-RP, and (d) Cu-RP. The iso-value has been set as 0.1.

**Supplementary Table 1.** Average Mulliken charge of Fe-RP, Co-RP, Ni-RP, and Cu-RP.

|  | Fe-RP | Co-RP | Ni-RP | Cu-RP |
| --- | --- | --- | --- | --- |
| Average Net Charge | **0.27 e** | **0.24 e** | **0.18 e** | **0.30 e** |
